# The effect of melatonin on bacterial translocation following ischemia/reperfusion injury in a rat model of superior mesenteric artery occlusion

**DOI:** 10.1186/s12893-015-0003-7

**Published:** 2015-03-08

**Authors:** Murat Ozban, Cagatay Aydin, Nural Cevahir, Cigdem Yenisey, Onur Birsen, Gulistan Gumrukcu, Berrin Aydin, Ibrahim Berber

**Affiliations:** Department of General Surgery, School of Medicine, Pamukkale University, Denizli, Turkey; Department of Microbiology, School of Medicine, Pamukkale University, Denizli, Turkey; Department of Biochemistry, School of Medicine, Adnan Menderes University, Aydin, Turkey; Department of Pathology, Haydarpasa Numune Training and Research Hospital, Istanbul, Turkey; Department of Emergency Medicine, Denizli State Hospital, Denizli, Turkey; Department of Transplantation, International Hospital, Istanbul, Turkey

**Keywords:** Melatonin, Ischemia-reperfusion, Bacterial translocation

## Abstract

**Background:**

Acute mesenteric ischemia is a life-threatening vascular emergency resulting in tissue destruction due to ischemia-reperfusion injury. Melatonin, the primary hormone of the pineal gland, is a powerful scavenger of reactive oxygen species (ROS), including the hydroxyl and peroxyl radicals, as well as singlet oxygen, and nitric oxide. In this study, we aimed to investigate whether melatonin prevents harmful effects of superior mesenteric ischemia-reperfusion on intestinal tissues in rats.

**Methods:**

Rats were randomly divided into three groups, each having 10 animals. In group I, the superior mesenteric artery (SMA) was isolated but not occluded. In group II and group III, the SMA was occluded immediately distal to the aorta for 60 minutes. After that, the clamp was removed and the reperfusion period began. In group III, 30 minutes before the start of reperfusion, 10 mg/kg melatonin was administered intraperitonally. All animals were sacrified 24 hours after reperfusion. Tissue samples were collected to evaluate the I/R-induced intestinal injury and bacterial translocation (BT).

**Results:**

There was a statistically significant increase in myeloperoxidase activity, malondialdehyde levels and in the incidence of bacterial translocation in group II, along with a decrease in glutathione levels. These investigated parameters were found to be normalized in melatonin treated animals (group III).

**Conclusion:**

We conclude that melatonin prevents bacterial translocation while precluding the harmful effects of ischemia/reperfusion injury on intestinal tissues in a rat model of superior mesenteric artery occlusion.

## Background

Acute mesenteric ischemia (AMI) is a life-threatening vascular emergency with an overall mortality of 60% to 80%, and besides its potential fatality, the incidence of this challenging surgical problem is reported to be increased [1]. Appropriate management of AMI requires early diagnosis and rapid intervention with therapeutic methods that adequately restore mesenteric blood flow to prevent bowel necrosis [2]*.* The estimated proportion of common forms of acute mesenteric ischemia is approximately 50% for superior mesenteric artery (SMA) embolism, followed by SMA thrombosis (25%), non-occlusive mesenteric ischaemia (20%) and mesenteric venous thrombosis (5%) [3]. Irrespective of the form of the mesenteric ischemia, the consequences are similar and include a range of intestinal tissue damage from mild disturbances in bowel function to transmural necrosis and gangrene [1]. This tissue destruction due to occlusion of mesenteric blood flow is often the result of cellular injury related to reperfusion following therapeutic interventions [4].

While short periods of mesenteric ischemia contribute to a mild increase in microvascular permeability, sustained ischemia and succeeding reperfusion results in disruption of the intestinal mucosal barrier, mainly due to generation of toxic oxygen free radicals, such as superoxide, peroxide, and hydroxyl radicals, which damage the cell membrane through lipid peroxidation [2]. The injured intestinal mucosa loses its resistance to indigenous enteric microorganisms, which leads to translocation of bacteria to extraintestinal sites such as mesenteric lymph nodes, liver, spleen, and blood [5]. This bacterial translocation may play an important role in the development of sepsis and multiple organ system failure [1].

The role of oxygen free radicals in reperfusion injury is demonstrated by the reduction of tissue damage in the presence of antioxidants and free-radical scavenging substances such as N-acetylcysteine, selenium, vitamins E and C, superoxide dismutase, and catalase [1]. Melatonin (*N*-acetyl-5-methoxytryptamine), the primary hormone of the pineal gland, is a powerful scavenger of reactive oxygen species (ROS), including the hydroxyl and peroxyl radicals, as well as singlet oxygen, and nitric oxide [6]. In addition to scavenging ROS, melatonin stimulates the antioxidant enzymes superoxide dismutase, glutathione peroxidase, and catalase [7,8]. Recently, several authors have described melatonin as one of the most effective antioxidants and scavengers of oxygen-free radicals after IR injury of the liver, lung, and intestine [8,9]. In this study, we aimed to investigate whether melatonin precludes bacterial translocation after intestinal ischemia-reperfusion injury in rats.

## Methods

Male Wistar-Albino rats weighing 250-300 g were used in this study. All animals were housed in a light-controlled room with a 12 hours light/dark cycle and were allowed free access to food and water. Animals were housed in the animal facility for at least 7 day prior to use to stabilize their intestinal flora. The operative procedure, use of anesthesia, and animal care methods in the experiments were consistent with the National Institutes of Health Guidelines on the Care and Use of Laboratory Animals (NIH publication No.86-23, revised 1985, Bethesda, MD) and also approved by the Experimental Animal Committee of Pamukkale University School of Medicine.

### Operative details

After fasting overnight, the rats were anesthetized by an intramuscular injection of ketamine 50 mg/kg (Ketalar; Parke Davis, Eczacibasi, Istanbul, Turkey) and xylazine 10 mg/kg (Rompun; Bayer AG, Leverkusen, Germany). Animals were allowed to breathe spontaneously during the surgery. A heating lamp was used to preserve the body temperature at approximately 37°C. At the end of the operation, 10 ml Ringer’s Lactated solution was administered subcutaneously, to prevent dehydration of the rats. The abdomen was shaved and twice soaked with 10% povidone-iodine solution before rats were aseptically operated using sterile instruments. After a midline laparotomy, the superior mesenteric artery (SMA) was exposed. At this stage of the experiment, rats were randomly divided into three groups, each having 10 animals. In group I (sham operated group), the superior mesenteric artery (SMA) was isolated but not occluded. In group II (intestinal I/R only group) and group III (intestinal I/R + melatonin treated group), the SMA was gently isolated, and occluded immediately distal to the aorta with collateral interruption for 60 minutes with atraumatic microvascular clamps as described elsewhere [10]. This procedure provided ischemia in small intestine, cecum and right colon which was confirmed when the mesenteric pulsation was lost and the intestines became pale. After 60 minutes of ischemia, the clamp was removed and the reperfusion period began. In group III, 30 minutes before the start of reperfusion, 10 mg/kg melatonin was administered intraperitonally. Subsequently, the abdominal incisions were closed in two layers with 3/0 polyglactin suture (Vicryl, Ethicon, UK). Animals were fed with standard rat chow and water postoperatively. All animals were anesthetized and euthanized 24 hours after reperfusion. Tissue samples were collected to evaluate the I/R-induced intestinal injury and bacterial translocation (BT). Using sterile technique and instruments, a midline laparotomy was performed to yield biopsies of liver, spleen, mesenteric lymph nodes, terminal ileum for quantitative culture of aerobic and anaerobic organisms. A 1 ml sample of blood from vena cava was taken and cultured in appropriate media for aerobic and anaerobic organisms. The segments of ileum were removed and frozen in liquid nitrogen and stored at –80°C for further biochemical analysis of lipid peroxidation, myeloperoxidase activity, and tissue glutathione (GSH) levels, and were also fixed in 10% formaldehyde solution for further histo-pathological examination.

### Microbiological analysis

Microbiological analysis was performed as described previously [11]. Blood (0.5 mL) samples were cultured in 5 mL of brain heart infusion broth for 7 days at 37°C. The cultures were inspected daily and subcultured on blood agar and eosine methylene blue (EMB) agar plates. Samples of mesenteric lymph nodes, liver, spleen and ileal contents were weighed and placed in a sterile grinding tube. Tissues were homogenized in 1 mL of saline for quantitative cultures. This preparation was plated on blood agar and EMB agar and was incubated at 37°C for 24-48 h in ambient air. The identification of bacterial species was performed by standard microbiologic methods. Colonization was expressed as the number of colony-forming units (CFU) per gram of tissue homogenate (CFU/g).

### Histopathological examinations

The sections of ileum embedded in Paraffin and they were stained with hematoxylin and eosin (H & E). A pathologist blinded to the experimental groups examined these sections under a light microscope. Intestinal mucosal lesions were graded on a scale from 0 to 5 as described by Chiu *et al.* [12]: grade 0, normal mucosal villi; grade 1, development of subepithelial Gruenhagen’s space, usually at the apex of the villus, often with capillary congestion; grade 2, extension of the subepithelial space with moderate lifting of the epithelial layer from the lamina propria; grade 3, massive epithelial lifting with a few denuded villi; grade 4, denuded villi with exposed dilated capillaries; and grade 5, digestion and disintegration of lamina propria, hemorrhage, and ulceration.

### Estimation of neutrophil accumulation

The levels of myeloperoxidase (MPO) activity in the segment of ileum were determined as an indicator of neutrophil accumalation. Tissues were homogenized in 50 mM phosphate buffer (pH 7.4) (1/10, w/v) containing protease inhibitor, 0.2 μM phenylmethanesulphonyl fluoride (PMSF) and 1 mM Ethylenediamin tetra acetic acid (EDTA), at 4°C for 30 s using a homogenizer (Potter S, B. Braun, Germany). Then, appropriate volume of homogenate was used MPO determination.

The method of Suzuki et al. was used with a slight modification [13]. This method is based on the oxidation of the synthetic substrate 3,3’,5,5’-tetramethyl benzidine (TMB) by MPO. The standard reaction mixture consisted of 500 μl detergent-containing buffer (160 mM potassium phosphate buffer, pH 5.4, 1% hexadecyltrimethylammonium bromide), 100 μl TMB (16 mM, dissolved in dimethylformamid), 50 μl homogenate and 300 μl water. The reaction was started by the addition of 50 μl H_2_O_2_ (diluted 0.003%) at 37°C. The rate of MPO-catalyzed oxidation of TMB was followed by recording the increase in absorbance at 655 nm. Considering the initial and linear phase of the reaction, we measured the absorbance change per minute, and one enzyme unit was defined as the amount of enzyme producing one absorbance change per minute under assay conditions. Enzyme activity was calculated as units per gram of wet weight tissue.

### Determination of lipid peroxidation

The levels of malondialdehyde (MDA) in ileal tissue specimens were determined as an indicator of lipid peroxidation. The MDA production and hence lipid peroxidation were assessed in the tissues by the method of Ohkowa. MDA forms a colored complex in the presence of TBA, which is detectable by measurement of absorbance at 532 nm. Absorbance was measured with Shimadzu UV-160 spectrophotometer. 1,1′,3,3′ -Tetraethoxypropane was used as a standard and the results were expressed in tissue as μmol/g protein [14].

### Determination of glutathione

The concentrations of glutathione (GSH) in tissue samples were measured by the method of Ellman [15]. One ml tissue homogenate precipitated by 2 ml of 5% TCA was taken and 0.5 ml of Ellman’s reagent (0.0198% DTNB in 1% sodium citrate) and 3 ml of phosphate buffer (pH 8.0) were added. The color developed was read at 412 nm. The concentration was expressed in mg/g protein in tissue samples.

### Statistics

The results were expressed as mean ± SEM. Statistical evaluation for proportional comparisons for positive cultures of tissues was made with Chi-square (Fisher’s Exact test) analysis. Comparisons for quantitative culture and ileal MDA, MPO, GSH levels were analyzed with Kruskal-Wallis test, and multiple comparisons between the groups were performed with Mann Whitney-U test. Differences were considered statistically significant when *P* <0.05. Data were analyzed by statistical software (SPSS for Windows 11.5; SPSS, Chicago, Illinois).

## Results

All of the animals survived the experiment period. We did not observe any side effect that can be attributable to melatonin during the study period.

### Bacterial translocation

The incidence of bacterial translocation to the mesenteric lymph nodes (MLN) (*P* = 0.0001), liver (*P* = 0.008), and spleen (*P* = 0.036) was significantly higher in I/R only animals (group II) than in sham operated and melatonin treated animals (groups I and III) (Table 1). Furthermore, the incidence of translocation was not statistically different in melatonin treated group from the value measured in sham operated group of animals for each location (*P* = 0.14 for MLN, *P* = 0.14 for liver, *P* = 1.00 for spleen). Bacterial translocation to blood was seen in only one animal that belongs to group II. The most common translocated microorganisms were Escherichia coli, klebsiella spp and enterobacter spp.

**Table 1:** **Incidence and number of bacterial translocation in tissue specimens**

**Groups (n = 10)**	**MLNs iIncidence**	**CFU ± SEM**	**Liver incidence**	**CFU ± SEM**	**Spleen incidence**	**CFU ± SEM**
Sham	0/10	-	0/10	-	0/10	-
I/R	8/10*	54050 ± 15315.65	6/10**	1075.5 ± 623.24	3/10***	80 ± 46.66
I/R + melatonin	2/10	3600 ± 2993.32	2/10	250 ± 200,69	2/10	-

(*P = 0.0001, **P = 0.008, ***P = 0.036 I/R versus Sham and I/R + melatonin).

### Ileal bacteria counts

Ileal counts of bacteria in sham operated group were significantly less from I/R and I/R + melatonin groups (*P* = 0.0001). Furthermore, melatonin treatment provided a significant reduction in ileal bacteria count in group III than in group II (I/R only) animals (*P* = 0.028) (Table 2).

**Table 2:** **Ileal bacteria counts**

**Groups**	**Ileal bacteria count**
Sham	60200 ± 13573.50*
I/R	419500 ± 49857.85
I/R = melatonin	247500 ± 48369.24

(*p = 0.0001).

### Histopathological examinations

In sham-operated animals (Group I), histopathological examinations of ileal epithelium and mucosal villi were normal. The specimens from this group were classified as grade 0–1 according to the Chiu’s intestinal mucosal injury scale. In I/R-only Group II (control group), grade 1–4 histopathological damage was detected. In I/R + melatonin treated Group III, grade 0–2 damage was detected. The median intestinal mucosal injury score in I/R-only Group II was significantly higher than that in sham-operated rats (Group I) (*P =* 0.0001), and also than that in melatonin-treated rats (Group III) (*P =* 0.003) (Table 3). In addition, the improvement in the intestinal mucosal injury score in I/R + melatonin-treated rats reached the same level for that of sham-operated Group I (*P =* 0.053) (Table 3). These results show that melatonin treatment significantly attenuated the intestinal mucosal villous injury.

**Table 3:** **Intestinal mucosal injury scores of the groups**

	**Sham**	**I/R**	**I/R + Tempol**
Intestinal mucosal injury scores	0 (0-1)	2 (1-4)	0 (0-2)*

Results were given as median (min-max).

(*P = 0, 3 versus I/R only Group II, P = 0, 053 versus sham-operated Group I).

### Effects of melatonin on tissue neutrophil accumulation

**Figure 1:**
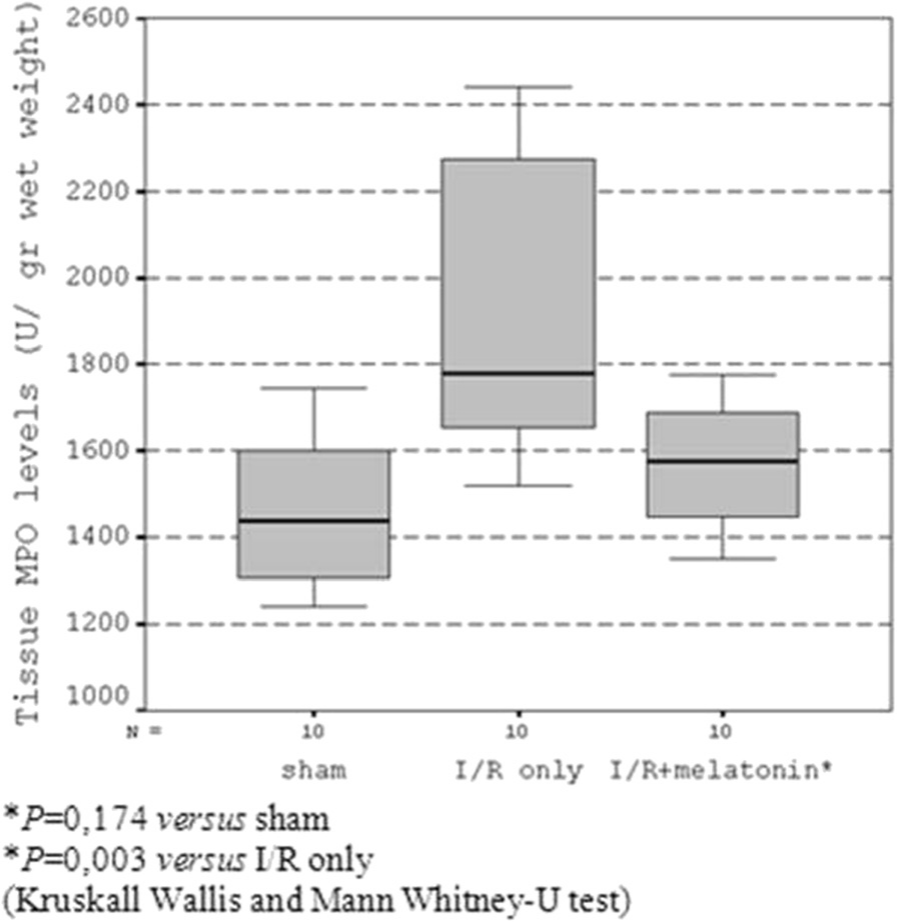
**Tissue MPO levels.**

When compared to sham group and I/R + melatonin group, group II rats exhibited a significant increase in the ileal tissue levels of MPO (*P* = 0.003). Treatment of rats with melatonin in group III resulted in a significant decrease in the levels of MPO in the ileal tissue (*P* = 0.013, group III vs group II, Figure 1).

### Effects of melatonin on intestinal ischemia reperfusion

#### GSH levels

**Figure 2:**
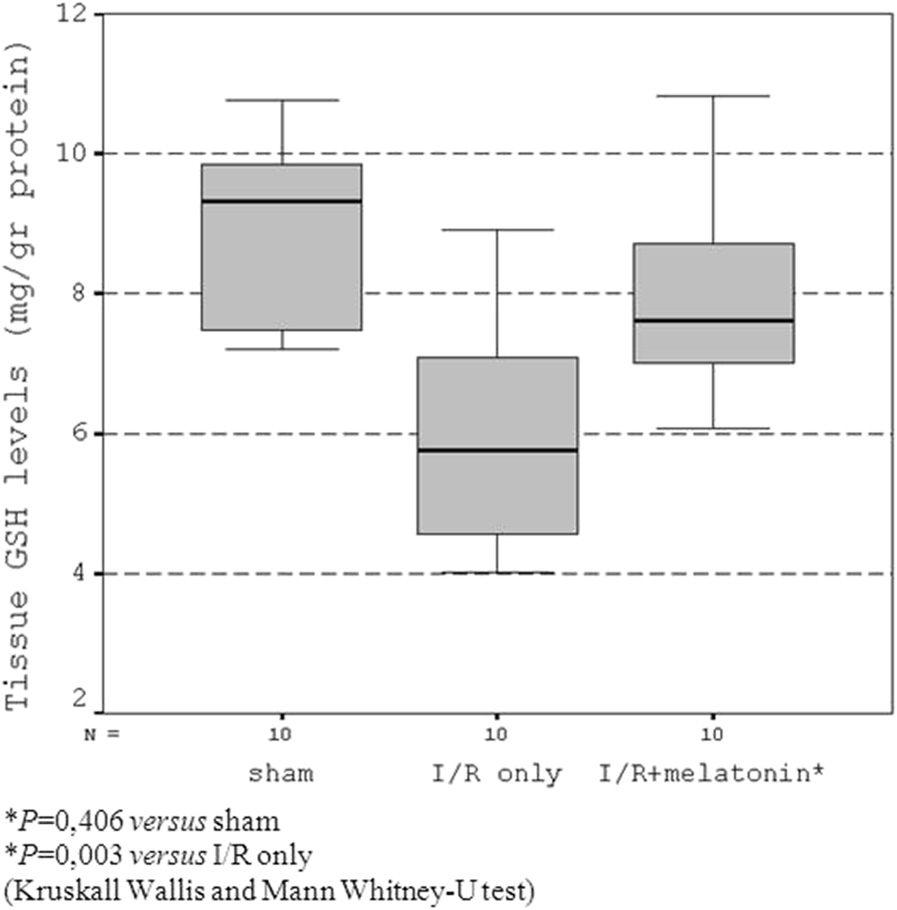
**Tissue GSH levels.**

The amount of GSH measured in intestinal tissues subjected to I/R injury alone was significantly reduced when compared to that in group I (sham) and I/R + melatonin groups (*P* = 0.003, Figure 2).

Administration of melatonin significantly ameliorated the decreased amount of GSH (*P* = 0.01 group II vs group III). The amount of GSH in group III (I/R + melatonin) was not different statistically from the value measured in the control group (*P* = 0.406).

#### MDA levels

**Figure 3:**
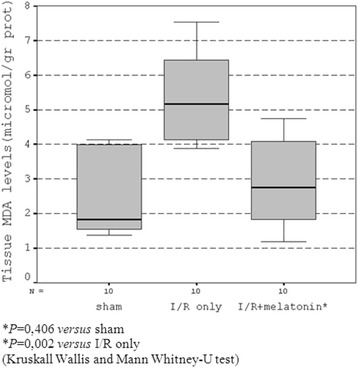
**Tissue MDA levels.**

The mean ileal tissue MDA levels were statistically different among the groups (*P* = 0.002, Figure 3). The mean MDA concentration in the intestinal tissue in group II was significantly higher than that in groups I and III at the end of the experiment. The amount of MDA in group III (I/R + melatonin) was not different statistically from the value measured in the sham operated group (*P* = 0.406).

## Discussion

According to the results of the present study, melatonin, the primary hormone of the pineal gland prevented intestinal bacterial translocation which was induced by mesenteric ischemia-reperfusion injury. This beneficial effect of melatonin seemed to be due to the ability of this agent to function as an intracellular scavenger of radical oxygen species (ROS) demonstrated by a reduction in the degree of lipid peroxidation, neutrophil accumulation and maintaining of GSH in ileal tissue specimens.

Physiologically, intestinal barrier function is composed of mucosal immunity and physical integrity [16]. The main mission of the epithelium overlying mucosal surfaces of the intestinal tract is providing an effective barrier to the microorganisms present in the intestinal lumen. However, it has become obvious recently that the epithelial layer is much more than a simple physical barrier [16]. The process of accomodating the microorganisms involves various host defence mechanisms which have evolved to regulate the composition of the them, and protection against infection and colonisation. The primary mediators of this activity are various anti-microbial peptides that are secreted by the epithelium and the mucus layer that coats the lumen. Below the mucus layer, the epithelial cells separated by junctions that represent binding of tight junction proteins [16]. The most abundant constituents of epithelial cellular membranes are phospholipids. When phospholipids are dispersed in water, they spontaneously form lipid bilayers. The lipid bilayer serves as a matrix for embedded proteins, which function as transporters, ion channels, receptors for hormones and neurotransmitters, etc. After ischemia/reperfusion injury, the oxygen radicals which are the products of this oxidative stress, destroys these lipid bilayers via lipid peroxidation [6]. Consequently, intestinal permeability increases and intestinal barrier function fails due to destruction of endothelial and epithelial cells resulting in breakdown of mucosal integrity [17]. Finally, indigenous enteric bacteria translocate to extraintestinal sites such as mesenteric lymph nodes, liver, spleen, and blood, resulting in sepsis and multiple organ system failure [17]. Since melatonin reduces lipid peroxidation in every cell and tissue, it was assumed that, in doing so, this hormone would also maintain cell membranes in a state of optimal fluidity and membrane rigidity [6]. Moreover, it has recently reported that derivatives of melatonin that are formed when the indoleamine functions as a scavenger may actually be more effective than melatonin itself in neutralizing the peroxyl radicals [18]. In our study, we observed a significantly reduced incidence of bacterial translocation to the blood, liver, spleen, and mesenteric lymph nodes in animals pretreated with melatonin in an acute mesenteric ischemia/reperfusion model. Furthermore, melatonin treatment provided a statistically significant reduction in ileal bacterial overgrowth. It was previously shown that mesenteric ischemia/reperfusion not only produces mucosal damage but also induces alterations of intestinal motor activity with a delay in gastrointestinal transit time [19]. These alterations can be the result of structural and neuronal changes occurring within the enteric nervous system which may play a role in the progress of bacterial overgrowth with subsequent translocation due to inadequate bacterial clearance [20]. In addition to its properties against I/R oxidative injury, melatonin has been suggested to act as a local regulator of gastrointestinal motility [21].

The administration of melatonin significantly decreased the histological damage (Table 3) and PMNs infiltration in intestinal tissue specimens in our study when compared with the I/R only group of animals (Group II). In a previous study, melatonin has been found to reduce histological signs of intestinal injury in rats subjected to splanchnic artery occlusion shock [22]. Melatonin also reduces the migration of PMNs into the inflammatory site [22]. This effect of melatonin seems probably secondary to the protection of intestinal mucosa to endothelial oxidative injury and therefore conservation of endothelial barrier function. Myeloperoxidase activity, one of the markers of neutrophil accumulation, was found to be elevated in intestinal I/R injury [23]. In our experiment, the reduction in neutrophil accumulation shown by a significant decrease in MPO activity within the ileal tissue of melatonin-treated group of animals is also seems to be secondary to the prevention of oxidative damage. The systemic activation of neutrophils after reperfusion appears to be secondary to mediators such as cytokines and ROS [24]. This activated neutrophils further promotes inflammation and oxidative damage. It was reported that this vicious cycle among I/R injury, endothelial damage, and neutrophil infiltration causes additional ROS production [25]. Previously, research has also demonstrated melatonin’s anti-inflammatory properties via down-regulation of proinflammatory cytokines [26]. The inhibitory effect of melatonin against PMN infiltration was also observed in another study in which oxidative stress was induced by ischemia and reperfusion [22]. When considered together with our findings, it seems that melatonin treatment effectively prevents I/R related intestinal injury by interfering with this vicious cycle.

Intestinal ischemia is a life-threatening abdominal emergency. The common clinical feature of the disease is caused by impaired blood perfusion of the intestine and the hypoxia associated sequelae, like bacterial translocation as well as local and systemic inflammation [1]. Thus, rapid restoration of mesenteric blood flow with reoxygenation of the ischemic tissue is critical to its salvage, but it may paradoxically exacerbate tissue damage [3]. During ischemia, there is an increase in microvascular permeability, release of lysosomal hydrolases and enhance in proteolysis [19]. Those alterations are aggravated by reperfusion, since it triggers the accumulation of free radicals, which attack and damage the cellular membranes, attract neutrophils, and stimulate the release of inflammatory mediators [17]. So in the clinical scenario of a patient suffering from acute mesenteric ischemia, a potentially therapeutic agent that is expected to block the deleterious effects of reperfusion such as release of free oxygen radicals, should be given before restoring the arterial flow. Therefore in our experimental model, melatonin was administered 30 minutes before the start of reperfusion in the treatment group (Group III), aiming that it is existed in circulation of the animal. Adverse effects of melatonin are few and it is generally regarded as safe in recommended dosages. There are isolated case reports of psychomotor disturbances (disorientation, fatigue, headache, dizziness, etc.), increased seizure risk, and blood clotting abnormalities associated with melatonin alone or in combination with other medications [27]. In experimental studies, melatonin doses up to 800 mg/kg failed to cause death in mice [6]. A lethal dose in 50% of mice, that is, LD50, has not been determined [6]. In humans, for most non-sleep related disorders, doses from 10-50 mg daily have been used safely and effectively [27]. We used a 10 mg/kg melatonin dosage in our study 30 minutes before reperfusion starts like other researchers did in experimental ischemia/reperfusion rat model investigations of melatonin [7,9,26]. It seems to be a reasonable amount and timing when the relatively short serum half-life (30-60 minutes) and the total amount of melatonin in the gastrointestinal tractus (up 400 times more than in the pineal gland) taken into account [27,28].

Numerous mechanisms have been implicated in the evolution and development of intestinal I/R injury. These are overproduction of reactive oxygen species, increased expression and infiltration of leukocytes, and production of inflammatory mediators such as cytokines [5]. Evidence has accumulated that melatonin is both a direct free radical scavenger and an indirect antioxidant because of its ability to promote the activities of a variety of antioxidative enzymes [29]. While it is clearly a lipid soluble agent, it seems also capable of entering the aqueous environments of the cell which allows melatonin to be protective of membranes from free radical damage [6]. ROS and other free oxygen radicals are believed to cause cellular injury and further necrosis via different mechanisms including especially peroxidation of cellular membrane lipids [5]. It has been previously showed in a rat model of splanchnic artery occlusion and reperfusion that melatonin treatment abolished the increase in lipid peroxidation products, probably in part by scavenging the very reactive hydroxyl and peroxyl radicals [22]. In this current experimental model, the occlusion of superior mesenteric artery followed by reperfusion resulted in a considerable increase in the ileal tissue levels of malondialdehyde, and melatonin treatment significantly prevented lipid peroxidation, which was verified by decreased MDA levels in the ileal tissues of animals.

GSH is an endogenous antioxidant found naturally in all animal cells. It has the potential of reacting with free radicals ensuing secondarily to I/R, and the provisions of glutathione precursors are protective for different types of free-radical-mediated cellular injury [30]. Oxidants can upregulate the transcription of gamma-glutamylcysteine synthase genes, which is a rate-limiting enzyme for the synthesis of GSH [31]. Melatonin’s high efficacy in reducing oxidative damage may involve both receptor-independent as well as receptor-mediated processes. In addition to direct free radical scavenging, antioxidative functions of melatonin may include synergistic actions with classic antioxidants and stimulation of the synthesis of the important intracellular antioxidant GSH [32]. Melatonin stimulates glutathione peroxidase which converts hydroperoxides, including H2O2, to water and oxygen while oxidizing GSH [29]. Once the oxidized form of glutathione is formed, it is recycled to GSH by glutathione reductase, another enzyme whose activity is enhanced by melatonin [30]. In this present study, the reduction of GSH in the ileal tissue specimens seems to be a result of oxidant injury, and the restoration of GSH in ileal tissues of melatonin-treated animals can be attributed to these antioxidant features of melatonin.

## Conclusion

In conclusion, this study demonstrates that melatonin significantly prevents the detrimental effects of ischemia-reperfusion injury on the intestinal tissue in a rat model of superior mesenteric artery occlusion. It can be speculated that these beneficial effects of melatonin can be mainly attributed to its antioxidant properties. Other findings of this study such as decreased neutrophil activation and improvement of bacterial translocation seem therefore to be secondary to those antioxidative effects. Ultimately, we believe that further clinical studies are needed to reveal the effectiveness of melatonin as a therapeutic agent in the clinical setting in mesenteric I/R injury.

## References

[1] Yasuhara H (2005). Acute mesenteric ischemia: the challenge of gastroenterology. Surg Today.

[2] Oldenburg WA, Lau LL, Rodenberg TJ, Edmonds HJ, Burger CD (2004). Acute mesenteric ischemia: a clinical review. Arch Intern Med.

[3] Renner P, Kienle K, Dahlke MH, Heiss P, Pfister K, Stroszczynski C (2011). Intestinal ischemia: current treatment concepts. Langenbecks Arch Surg.

[4] Schoenberg MH, Beger HG (1993). Reperfusion injury after intestinal ischemia. Crit Care Med.

[5] Kong SE, Blennerhassett LR, Heel KA, McCauley RD, Hall JC (1998). Ischaemia-reperfusion injury to the intestine. Aust N Z J Surg.

[6] Garcia JJ, Lopez-Pingarron L, Almeida-Souza P, Tres A, Escudero P, Garcia-Gil FA (2014). Protective effects of melatonin in reducing oxidative stress and in preserving the fluidity of biological membranes: a review. J Pineal Res.

[7] Ding K, Wang H, Xu J, Li T, Zhang L, Ding Y (2014). Melatonin stimulates antioxidant enzymes and reduces oxidative stress in experimental traumatic brain injury: the Nrf2-ARE signaling pathway as a potential mechanism. Free Radic Biol Med.

[8] Singh M, Jadhav HR (2014). Melatonin: functions and ligands. Drug Discov Today.

[9] Takhtfooladi H, Takhtfooladi M, Moayer F, Mobarakeh S. Melatonin attenuates lung injury in a hind limb ischemia-reperfusion rat model. Rev Port Pneumol 2014. doi:10.1016/j.rppneu.2014.01.007. [Epub ahead of print]. 10.1016/j.rppneu.2014.01.007 24661959

[10] Kuzu MA, Tanik A, Kale IT, Aslar AK, Koksoy C, Terzi C (2000). Effect of ischemia/reperfusion as a systemic phenomenon on anastomotic healing in the left colon. World J Surg.

[11] Isenberg H (1992). Clinical microbiology procedures handbook, vol. 1.

[12] Chiu CJ, McArdle AH, Brown R, Scott HJ, Gurd FN (1970). Intestinal mucosal lesion in low-flow states. I. A morphological, hemodynamic, and metabolic reappraisal. Arch Surg.

[13] Suzuki K, Ota H, Sasagawa S, Sakatani T, Fujikura T (1983). Assay method for myeloperoxidase in human polymorphonuclear leukocytes. Anal Biochem.

[14] Ohkawa H, Ohishi N, Yagi K (1979). Assay for lipid peroxides in animal tissues by thiobarbituric acid reaction. Anal Biochem.

[15] Ellman GL (1959). Tissue sulfhydryl groups. Arch Biochem Biophys.

[16] Man AL, Gicheva N, Nicoletti C (2014). The impact of ageing on the intestinal epithelial barrier and immune system. Cell Immunol.

[17] Vollmar B, Menger MD (2011). Intestinal ischemia/reperfusion: microcirculatory pathology and functional consequences. Langenbecks Arch Surg.

[18] Galano A (2011). On the direct scavenging activity of melatonin towards hydroxyl and a series of peroxyl radicals. Phys Chem Chem Phys.

[19] Pontell L, Sharma P, Rivera LR, Thacker M, Tan YH, Brock JA (2011). Damaging effects of ischemia/reperfusion on intestinal muscle. Cell Tissue Res.

[20] Lindestrom LM, Ekblad E (2004). Structural and neuronal changes in rat ileum after ischemia with reperfusion. Dig Dis Sci.

[21] Siah KT, Wong RK, Ho KY (2014). Melatonin for the treatment of irritable bowel syndrome. World J Gastroenterol.

[22] Cuzzocrea S, Costantino G, Mazzon E, Micali A, De Sarro A, Caputi AP (2000). Beneficial effects of melatonin in a rat model of splanchnic artery occlusion and reperfusion. J Pineal Res.

[23] Aydin C, Teke Z, Aytekin F, Yenisey C, Kabay B, Simsek NG (2007). Tempol prevents harmful effects of remote ischemia reperfusion injury on healing of experimental colonic anastomoses. Int J Colorectal Dis.

[24] Essani NA, Fisher MA, Farhood A, Manning AM, Smith CW, Jaeschke H (1995). Cytokine-induced upregulation of hepatic intercellular adhesion molecule-1 messenger RNA expression and its role in the pathophysiology of murine endotoxin shock and acute liver failure. Hepatology.

[25] Cuzzocrea S, McDonald MC, Mazzon E, Siriwardena D, Costantino G, Fulia F (2000). Effects of tempol, a membrane-permeable radical scavenger, in a gerbil model of brain injury. Brain Res.

[26] Ahsen A, Gonul Y, Genc A, Ulu MS, Yagmurca M, Kocogullari CU (2014). Protective effect of melatonin on infrarenal aortic occlusion: this effect is related to anti-inflammatory effect and antioxidant effect. Inflammation.

[27] Melatonin. Monograph. Altern Med Rev 2005, 10(4):326-36. 16366741

[28] Acuna-Castroviejo D, Escames G, Venegas C, Diaz-Casado ME, Lima-Cabello E, Lopez LC (2014). Extrapineal melatonin: sources, regulation, and potential functions. Cell Mol Life Sci.

[29] Rodriguez C, Mayo JC, Sainz RM, Antolin I, Herrera F, Martin V (2004). Regulation of antioxidant enzymes: a significant role for melatonin. J Pineal Res.

[30] Jefferies H, Coster J, Khalil A, Bot J, McCauley RD, Hall JC (2003). Glutathione. ANZ J Surg.

[31] Nakamura S, Sugiyama S, Fujioka D, Kawabata K, Ogawa H, Kugiyama K (2003). Polymorphism in glutamate-cysteine ligase modifier subunit gene is associated with impairment of nitric oxide-mediated coronary vasomotor function. Circulation.

[32] Reiter RJ, Tan DX, Terron MP, Flores LJ, Czarnocki Z (2007). Melatonin and its metabolites: new findings regarding their production and their radical scavenging actions. Acta Biochim Pol.

